# Supplementary material for: Serological Evidence That SARS-CoV-2 Has Not Emerged in Deer in Germany or Austria during the COVID-19 Pandemic
Source: Microorganisms. 2022 Mar 30;10(4):748. doi: 10.3390/microorganisms10040748 (PMC9031146; doi:10.3390/microorganisms10040748)
Supplement: Supplementary file 1 [file microorganisms-10-00748-s001.zip › microorganisms-1654981 Suppl final/microorganisms-1654981 Figure S1.pdf]

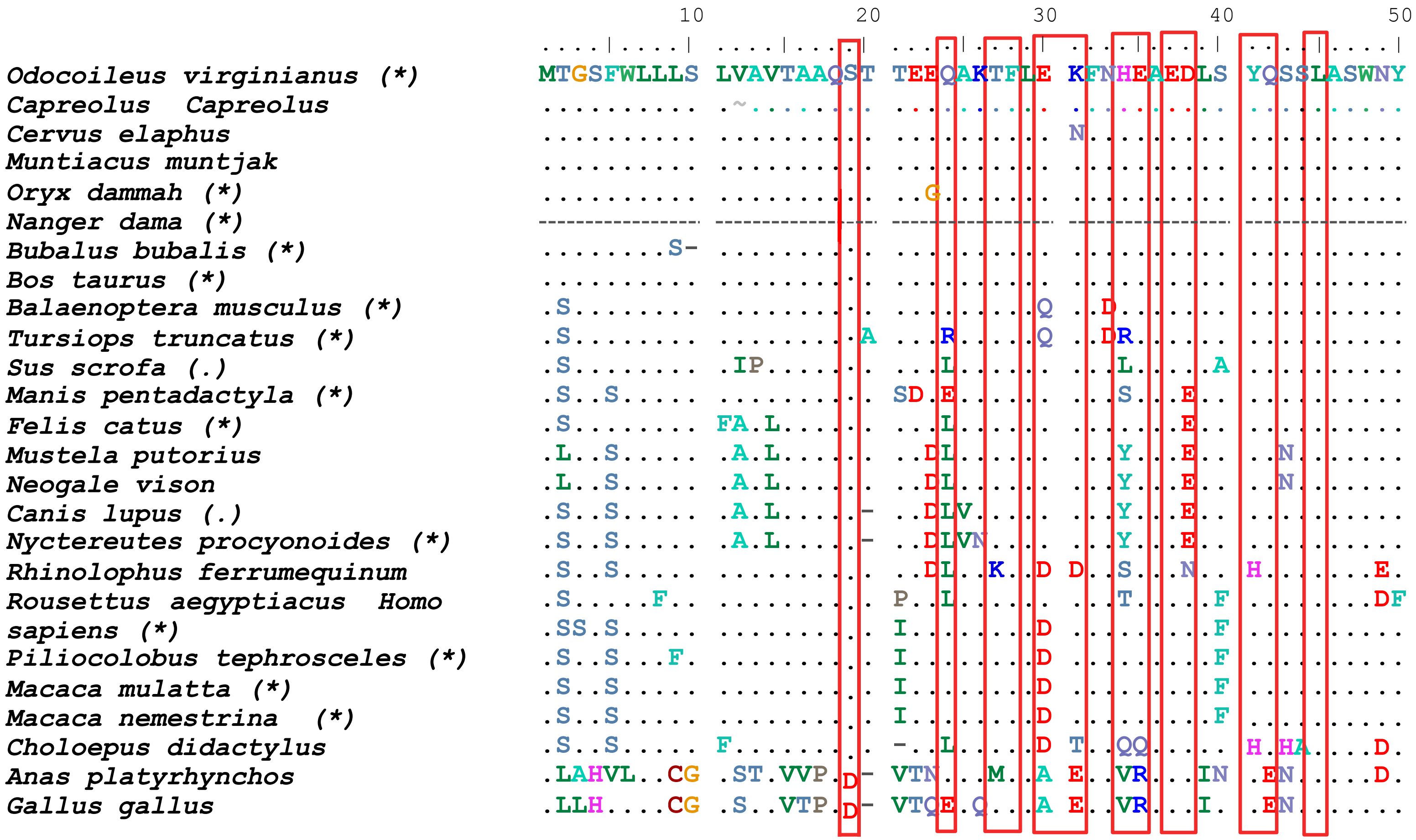

10
20
30
40
50
Odocoileus virginianus (*)
MTGSFWLLLS LVAVTAAQST TEEQAKTFLE KFNHEAEDLS YQSSLASWNY
Capreolus Capreolus
Cervus elaphus
Muntiacus muntjak
Oryx dammah (*)
Nanger dama (*)
Bubalus bubalis (*)
Bos taurus (*)
Balaenoptera musculus (*)
Tursiops truncatus (*)
Sus scrofa (.)
Manis pentadactyla (*)
Felis catus (*)
Mustela putorius
Neogale vison
Canis lupus (.)
Nyctereutes procyonoides (*)
Rhinolophus ferrumequinum
Rousettus aegyptiacus Homo sapiens (*)
Piliocolobus tephrosceles (*)
Macaca mulatta (*)
Macaca nemestrina (*)
Choloepus didactylus
Anas platyrhynchos
Gallus gallus

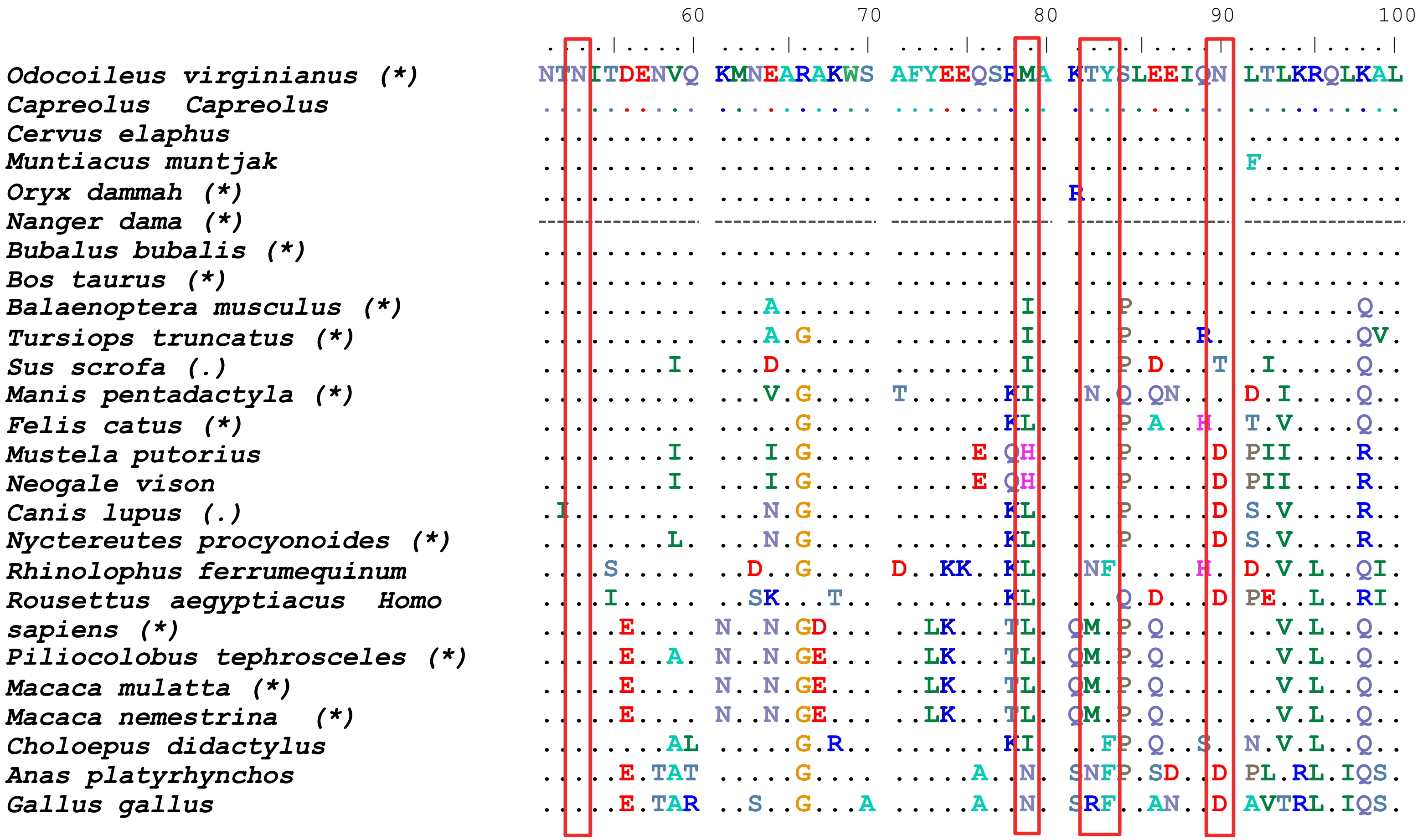
60
70
80
90
100
Odocoileus virginianus (*)
NTNITDENVQ KMNEARAKWS AFYEEQSRMA KTYSLEEIQN LTLKRQLKAL
Capreolus Capreolus
Cervus elaphus
Muntiacus muntjak
Oryx dammah (*)
Nanger dama (*)
Bubalus bubalis (*)
Bos taurus (*)
Balaenoptera musculus (*)
Tursiops truncatus (*)
Sus scrofa (.)
Manis pentadactyla (*)
Felis catus (*)
Mustela putorius
Neogale vison
Canis lupus (.)
Nyctereutes procyonoides (*)
Rhinolophus ferrumequinum
Rousettus aegyptiacus Homo sapiens (*)
Piliocolobus tephrosceles (*)
Macaca mulatta (*)
Macaca nemestrina (*)
Choloepus didactylus
Anas platyrhynchos
Gallus gallus

	101–110	111–120	121–130	131–140	141–150
Odocoileus virginianus (*)	QQSGTSVLSA	EKSKRLNTIL	NTMSTIYSTG	KVLDPN-TQE	CLALEPGLDD
Capreolus Capreolus	XX	XXXXX.....	....	......-...	....
Cervus elaphus	....	....	....	......-...	....
Muntiacus muntjak	....	....	....	......-...	....
Oryx dammah (*)	.H.......T	....	.K........	......-...	....
Nanger dama (*)	----------	----------	----------	----------	----------
Bubalus bubalis (*)	.H...A....	....Q.....	.K........	......-...	....
Bos taurus (*)	.H...A....	....	.K........	......-...	....
Balaenoptera musculus (*)	....	D.........	........S.	......-...	Y.V.......
Tursiops truncatus (*)	....	D......A..	S.......S.	......-...	S.V.......
Sus scrofa (.)	.....G....	D.........	........S.	......NP..	..V......E
Manis pentadactyla (*)	.L..S.A...	D.NQ......	....	..CN.GNP..	.SL......N
Felis catus (*)	....S.....	D..Q......	.A........	.ACN..NP..	..L.......
Mustela putorius	....S.....	D.RE......	.A........	.ACN..NP..	..L.......
Neogale vison	....S.....	D.RE......	.A........	.ACN..NP..	..L.......
Canis lupus (.)	.H..S.....	D.NQ......	.S...V....	.ACN.SNP..	..L.......
Nyctereutes procyonoides (*)	.H..S.....	D.NQ......	.S........	.ACN.SNP..	..L.......
Rhinolophus ferrumequinum	....SP...E	D......S..	.A........	..CK..NP..	..L......N
Rousettus aegyptiacus	....S.T...	D.T....D..	....	.ICQ..NS..	..L.......
Homo sapiens (*)	..N.S....E	D.........	....	..CN.DNP..	..L.....NE
Piliocolobus tephrosceles (*)	..N.S....E	D.........	....	..CN..NP..	..L.D...NE
Macaca mulatta (*)	..N.S....E	D.........	....	..CN..NP..	..L.D...NE
Macaca nemestrina (*)	..N.S....E	D.........	....	..CN..NP..	..L.D...NE
Choloepus didactylus	....S.....	D.N....I..	.A..S.....	..CNASNPK.	.FF.......
Anas platyrhynchos	.DK.S....P	..YS....V.	....	T.CKTTAPFD	.MV......S
Gallus gallus	.DR.S....P	..YS...SVM	.S........	V.CKATEPFD	..V.......

	160	170	180	190	200
	....|....|	....|....|	....|....|	....|....|	....|....|
Odocoileus virginianus (*)	IMENSRDYNR	RLWAWEGWRA	EVGKQLRPLY	EEYVVLENEM	ARANNYEDYG
Capreolus Capreolus	........S.	....	....	....	....X.....
Cervus elaphus	....	....	....	....	....
Muntiacus muntjak	....	....	....	....	....
Oryx dammah (*)	....	....	....	....	....
Nanger dama (*)	----------	----------	----------	----------	----------
Bubalus bubalis (*)	....	....	....	....	....
Bos taurus (*)	....	....	....	....	....
Balaenoptera musculus (*)	.....E....	....	........F.	....	....
Tursiops truncatus (*)	.....K....	....	....	....	....
Sus scrofa (.)	.....K..S.	......S...	....	....	....
Manis pentadactyla (*)	...S.K...E	.........S	....	......K...	....H.....
Felis catus (*)	.....K...E	....	....	....A.K...	....
Mustela putorius	.....K...E	.........S	....	....A.K...	....
Neogale vison	.....K...E	.........S	....	....A.K...	....
Canis lupus (.)	.....K...E	.........S	....	....A.K...	....
Nyctereutes procyonoides (*)	.....K...E	.........S	....	....A.K...	....
Rhinolophus ferrumequinum	...T.K...E	....	....	......K...	..GYH.....
Rousettus aegyptiacus *Homo*	...S.K..SQ	......S..S	........Y.	......K...	..GE......
sapiens (*)	..A..L...E	......S..S	....	......K...	....H.....
Piliocolobus tephrosceles (*)	...K.L...E	.........S	....	......K...	....H.K...
Macaca mulatta (*)	...K.L...E	.........S	....	......K...	.G..H.K...
Macaca nemestrina (*)	...K.L...E	.........S	....	......K...	....H.K...
Choloepus didactylus	........DE	.........S	.......L..	......K...	....
Anas platyrhynchos	..A..I..HE	....	D..RMM....	....D.K..A	.KL.G.A...
Gallus gallus	..A..I..HE	....	D..RMM....	....E.K..A	..L...S...

```
                                                      210        220        230        240        250
                                              ....|....| ....|....| ....|....| ....|....| ....|....|
Odocoileus virginianus (*)                    DYWRGDYEVT EAGDYDYSRD QLMKDVENTF AEIKPLYEQL HAYVRAKLMD
Capreolus  Capreolus                          .......... .......... .......H.. .......... ..........
Cervus elaphus                                .......... .......... .......H.. .......... ..........
Muntiacus muntjak                             .......... .......... .......H.. .......... ..........
Oryx dammah (*)                               .......... G......... .......R.. .......... ..........
Nanger dama (*)                               ---------- ---------- ---------- ---------- ----------
Bubalus bubalis (*)                           .......... G......... .......H.. .......... .........H
Bos taurus (*)                                .......... G......... .......R.. .......... .........H
Balaenoptera musculus (*)                     .......... G..V.....N ..IA...R.. .......... ..........
Tursiops truncatus (*)                        .......... G......... ..IR...R.. .......... ..........
Sus scrofa (.)                                .......... GT.......N ...E...R.. ........H. ..........
Manis pentadactyla (*)                        ........TE G.NG.N.... H.IE...HI. TQ......H. ..........
Felis catus (*)                               ........EE WTDG.N...S ..I....H.. TQ.....QH. ..........
Mustela putorius                              ........EE W.DG.S...N ..IE...H.. TQ......H. ..........
Neogale vison                                 ........EE W.DG.N...N ..IE...H.. TQ......H. ..........
Canis lupus (.)                               ........EE WENG.N...N ..ID...L.. TQ.M...QH. .....T....
Nyctereutes procyonoides (*)                  ........EE WENG.N...N ..ID...H.. TQ.M...QH. .....T....
Rhinolophus ferrumequinum                     ....R...TE GSP.LE.... ..I....RI. .......... .....T....
Rousettus aegyptiacus  Homo                   ........TE GINGSA.T.. ..IE..DRI. T......... .....T....
sapiens (*)                                   .........N GVDG.....G ..IE...H.. E.......H. .........N
Piliocolobus tephrosceles (*)                 ........AN GVDG...N.. ..IE...H.. E.......H. .........N
Macaca mulatta (*)                            .........N GVDG..NN.. ..IE...R.. E.......H. .........N
Macaca nemestrina  (*)                        .........N GVDG...N.. ..IE...R.. E.......H. .........N
Choloepus didactylus                          ........TE GENG.A.N.S ..I....H.. E.......H. .....T..T.
Anas platyrhynchos                            ....AN..AD YPEE.K.... ..IQ...K.. EQ.....Q.. .....HR.EQ
Gallus gallus                                 ....AN..TD YPEE.K.... ..VQ...K.. EQ.....QH. .....HR.EQ
```

```
                                       260        270        280        290        300
                                ....|....| ....|....| ....|....| ....|....| ....|....|
Odocoileus virginianus (*)      TY-PSYISPT GCLPAHLLGD MWGRFWTNLY SLTVPFKHKP SIDVTEKMKN
Capreolus  Capreolus            ..-....... .........X .......... .......... ........E.
Cervus elaphus                  ..-....... .......... .......... .......... ........E.
Muntiacus muntjak               A.-....... .......... .......... .......... ...I....E.
Oryx dammah (*)                 ..-....... .......... .......... ......E... ..........
Nanger dama (*)                 ---------- ---------- ---------- ---------- ----------
Bubalus bubalis (*)             ..-....... .......... .......... ......E... ........E.
Bos taurus (*)                  ..-....... .......... .......... ......E... ........E.
Balaenoptera musculus (*)       A.-..R.... .......... .......... P.....GE.. .....KE.Q.
Tursiops truncatus (*)          A.-..R.... .......... .......... P.....GER. .....KE.Q.
Sus scrofa (.)                  A.-..R.... .......... .......... P.....GE.. .......A.V.
Manis pentadactyla (*)          N.-..H.... .......... .......... P.....RQ.. N....DA.V.
Felis catus (*)                 ..-..R.... .......... .......... P.....GQ.. N....DA.V.
Mustela putorius                A.-..R.... .......... .......... P.M...RQ.. N....DA.V.
Neogale vison                   A.-..R.... .......... .......... P.M...GQ.. N....DA.V.
Canis lupus (.)                 ..-....... .......... .......... P.....GQ.. N....NA.V.
Nyctereutes procyonoides (*)    ..-....... .......... .......... P.....GQ.. N....NA.V.
Rhinolophus ferrumequinum       ..-.FH.... .......... .......... P.....GQ.. N....DA.L.
Rousettus aegyptiacus  Homo     A.-..H.... .......... ......I... P.....EQ.. N....DE.V.
sapiens (*)                     A.-......I .......... .......... ......GQ.. N....DA.VD
Piliocolobus tephrosceles (*)   A.-....... .......... .......... ......GQ.. N....DA.V.
Macaca mulatta (*)              A.-....... .......... .......... ......GQ.. N....DA.V.
Macaca nemestrina  (*)          A.-....... .......... .......... ......GQ.. N....DA.V.
Choloepus didactylus            F.-..H.... .......... .......... ......E... N....DE.VR
Anas platyrhynchos              A.GSQF..S. .......... .......... P....YPA.. N....DA.VQ
Gallus gallus                   V.GSEL.N.. .......... .......... N....YPE.. N....SA.AQ
```

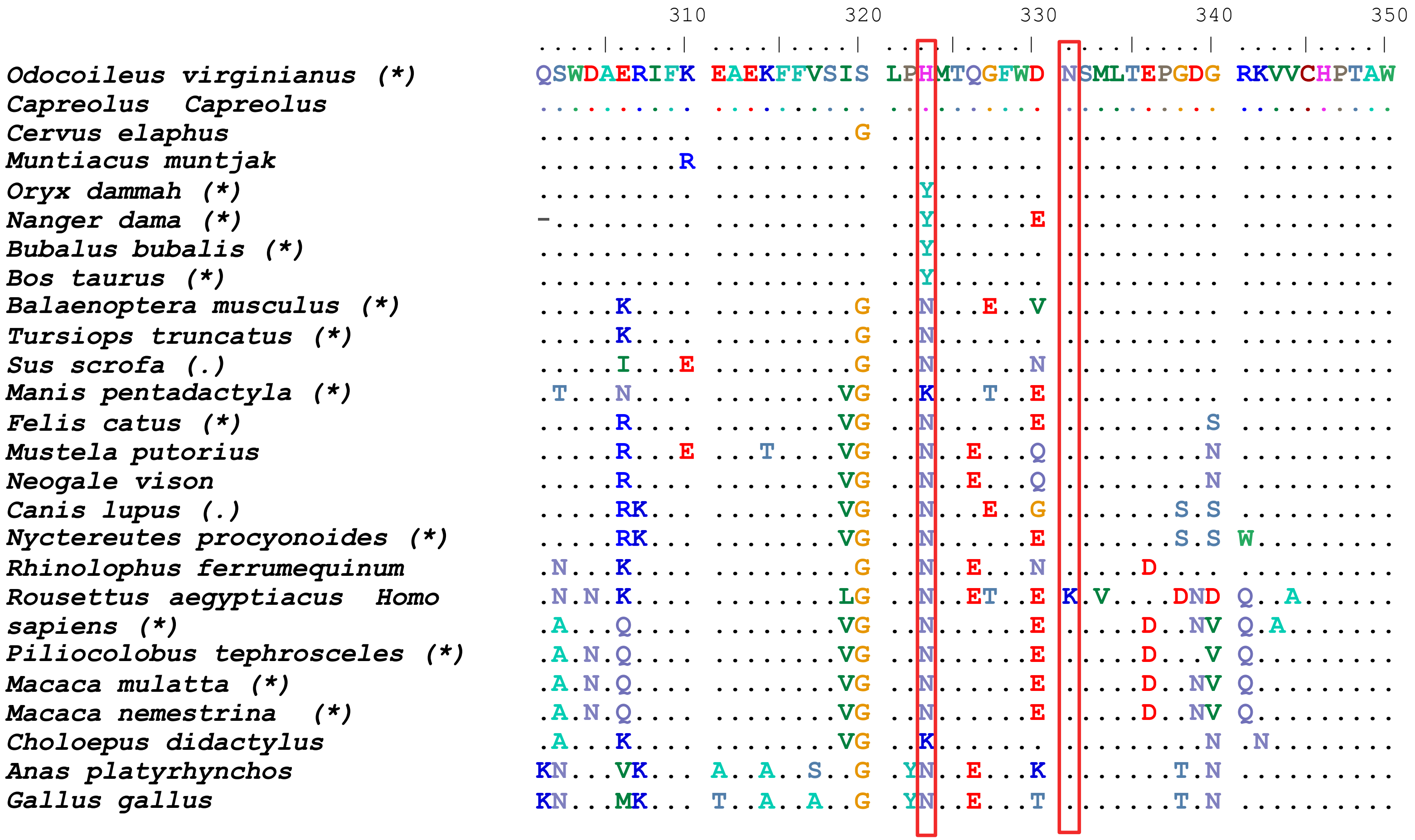
310
320
330
340
350
Odocoileus virginianus (*)
QSWDAERIFK EAEKFFVSIS LPHMTQGFWD NSMLTEPGDG RKVVCHPTAW
Capreolus Capreolus
Cervus elaphus
Muntiacus muntjak
Oryx dammah (*)
Nanger dama (*)
Bubalus bubalis (*)
Bos taurus (*)
Balaenoptera musculus (*)
Tursiops truncatus (*)
Sus scrofa (.)
Manis pentadactyla (*)
Felis catus (*)
Mustela putorius
Neogale vison
Canis lupus (.)
Nyctereutes procyonoides (*)
Rhinolophus ferrumequinum
Rousettus aegyptiacus Homo
sapiens (*)
Piliocolobus tephrosceles (*)
Macaca mulatta (*)
Macaca nemestrina (*)
Choloepus didactylus
Anas platyrhynchos
Gallus gallus

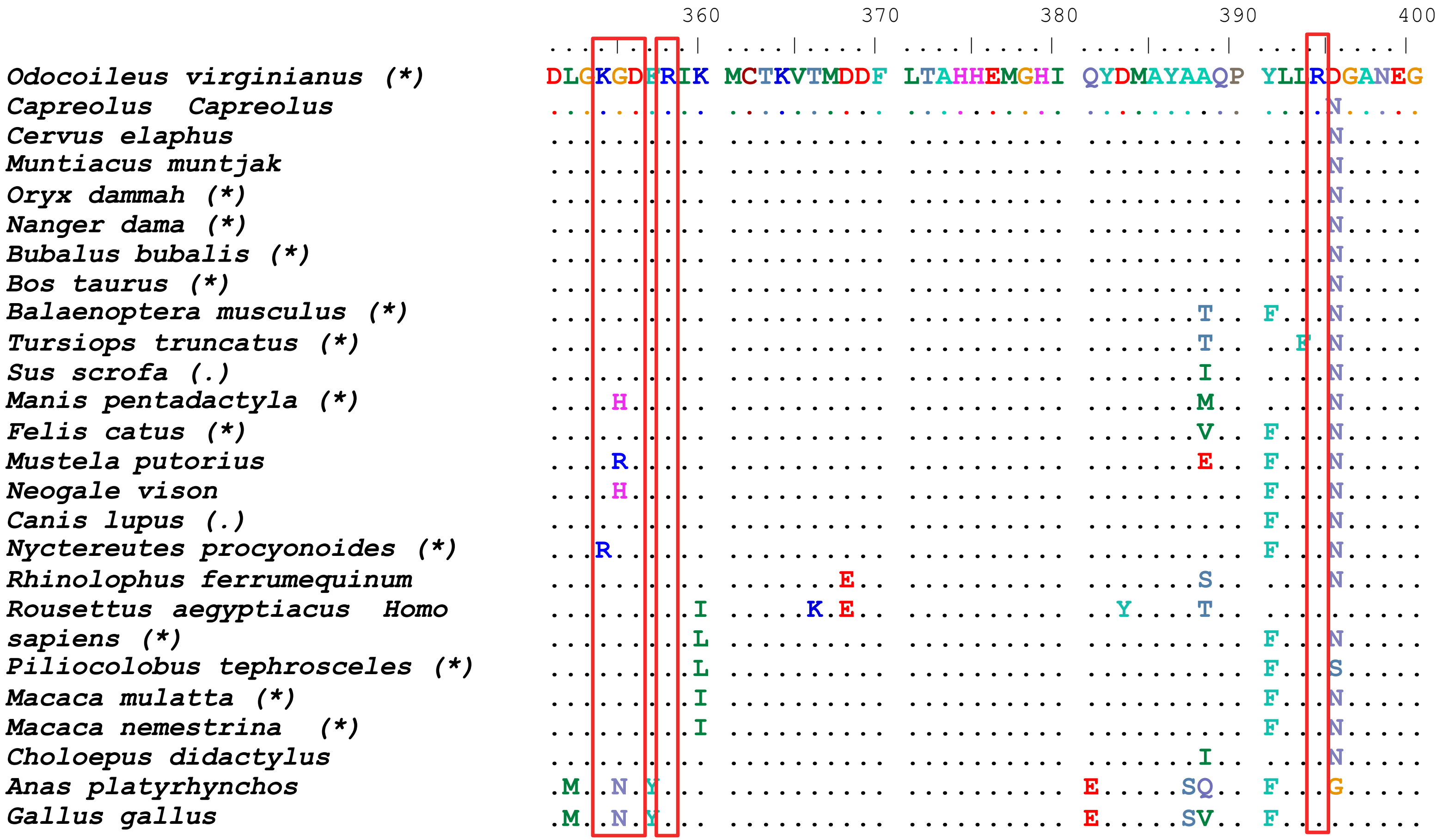
360
370
380
390
400
Odocoileus virginianus (*)
DLGKGDFRIK MCTKVTMDDF LTAHHEMGHI QYDMAYAAQP YLIRDGANEG
Capreolus Capreolus
Cervus elaphus
Muntiacus muntjak
Oryx dammah (*)
Nanger dama (*)
Bubalus bubalis (*)
Bos taurus (*)
Balaenoptera musculus (*)
Tursiops truncatus (*)
Sus scrofa (.)
Manis pentadactyla (*)
Felis catus (*)
Mustela putorius
Neogale vison
Canis lupus (.)
Nyctereutes procyonoides (*)
Rhinolophus ferrumequinum
Rousettus aegyptiacus Homo
sapiens (*)
Piliocolobus tephrosceles (*)
Macaca mulatta (*)
Macaca nemestrina (*)
Choloepus didactylus
Anas platyrhynchos
Gallus gallus

	410	420	430	440	450
Odocoileus virginianus (*)	FHEAVGEIMS	LSAATPHYLK	ALGLLEPDFY	EDNETEINFL	LKQALTIVGT
Capreolus Capreolus	....	....	....	....	....
Cervus elaphus	....	....	....	....	....
Muntiacus muntjak	....	....	....	....	....
Oryx dammah (*)	....	....	.....A....	....	....
Nanger dama (*)	....	....	.....A....	....	....
Bubalus bubalis (*)	....	....	.....A...H	....	....
Bos taurus (*)	....	....	.....A...H	....	....
Balaenoptera musculus (*)	....	....	.....P....	...V......	.....Q....
Tursiops truncatus (*)	....	....	.....P....	..SA......	....
Sus scrofa (.)	....	....	.....P....	..S.......	....
Manis pentadactyla (*)	....	......KH..	NI...P....	....	....
Felis catus (*)	....	......NH..	TI...S.G.S	..S.......	....
Mustela putorius	....	......NH..	NI...P...S	..S..D....	....
Neogale vison	....	......NH..	NI...P...S	..S..D....	....
Canis lupus (.)	....	......NH..	NI...P.S.F	..S.......	....
Nyctereutes procyonoides (*)	....	......NH..	NI...P.S.F	..S.......	....
Rhinolophus ferrumequinum	.......V..	..V...KH..	TM...SS..L	....	F....N....
Rousettus aegyptiacus	.......VI.	..V...NH..	NM...P....	....	.....NV...
Homo sapiens (*)	....	......KH..	SI...S...Q	....	....
Piliocolobus tephrosceles (*)	....	......KH..	SI...S...Q	....	....
Macaca mulatta (*)	....	......KH..	SI...S...Q	....	....
Macaca nemestrina (*)	....	......KH..	SI...S...Q	....	....
Choloepus didactylus	....	......KH..	.I...P...Q	..F.......	....
Anas platyrhynchos	....	......EH..	S.D....T.Q	..E.......	....
Gallus gallus	....	......QH..	S.D....T.Q	..E.......	....

	451–460	461–470	471–480	481–490	491–500
Odocoileus virginianus (*)	LPFTYMLEKW	RWMVFKGEIP	KEQWMEKWWE	MKREIVGVVE	PLPHDETYCD
Capreolus Capreolus	....	....	....	....	....
Cervus elaphus	....	....	.....Q....	....	....
Muntiacus muntjak	....	....	.....Q....	....	....
Oryx dammah (*)	....	....	.Q........	....	....
Nanger dama (*)	....	....	.Q........	....	....
Bubalus bubalis (*)	....	....	.Q........	....	....
Bos taurus (*)	....	....	.Q........	....	....
Balaenoptera musculus (*)	....	....	.....Q....	....	....
Tursiops truncatus (*)	....	....	.....Q....	....	....
Sus scrofa (.)	....	....	.....Q....	....	....
Manis pentadactyla (*)	....	.....S.Q..	.....K....	....	.V........
Felis catus (*)	....	....	.....Q....	....	.V........
Mustela putorius	....	....	.....Q....	...D......	....
Neogale vison	....	....	.....Q....	...D......	....
Canis lupus (.)	....	....	.D...KT...	...N......	.V........
Nyctereutes procyonoides (*)	....	....	.D...KT...	...N......	.V........
Rhinolophus ferrumequinum	....	....	..E..K....	...K......	.V........
Rousettus aegyptiacus Homo	....	....	....	....L.....	....
sapiens (*)	....	....	.D...K....	....	.V........
Piliocolobus tephrosceles (*)	....	.....E....	.D...K....	....	.V........
Macaca mulatta (*)	....	....	.D...K....	....	.V........
Macaca nemestrina (*)	....	....	.D...K....	....	.V........
Choloepus didactylus	....	.....R....	....TK....	..Q.....M.	.V....S...
Anas platyrhynchos	M.........	.....R...T	.QE.TKQ...	...D......	.V........
Gallus gallus	M.........	.....N...T	.QE.TKR..K	....	.V........

```
                                          510       520       530       540       550
                                  ....|....| ....|....| ....|....| ....|....| ....|....|
Odocoileus virginianus (*)        PACLFHVAED YSFIRYYTRT IYQFQFHEAL CKTANHEGAL FKCDISNSTE
Capreolus  Capreolus              .......... .......... .......... .......... ..........
Cervus elaphus                    .......... .......... .......... ....K..... ..........
Muntiacus muntjak                 .......... .......... .......... .......... ..........
Oryx dammah (*)                   .......... .......... .......... ....K..... ..........
Nanger dama (*)                   .......... .......... .......... ....K..... ..........
Bubalus bubalis (*)               .......... .......... .......... ....K..... ..........
Bos taurus (*)                    .......... .......... .......... ....K..... ..........
Balaenoptera musculus (*)         .......... .......... .......... .Q..K...P. Y.........
Tursiops truncatus (*)            .......... .......... .......... .Q..K...P. Y.........
Sus scrofa (.)                    .......... .......... .......... .R..K...P. Y.........
Manis pentadactyla (*)            ..S.....N. .......... ......Q... .Q..K...P. H.........
Felis catus (*)                   ..S.....N. .......... ......Q... .RI.K...P. H.......S.
Mustela putorius                  ..A.....N. .......... ......Q... .QI.K...P. Y.......S.
Neogale vison                     ..A.....N. .......... ......Q... .QI.K...P. Y.......R.
Canis lupus (.)                   ..S.....N. .......... ......Q... .QI.K...P. H.......S.
Nyctereutes procyonoides (*)      ..S.....N. .......... ......Q... .QI.K...P. H.......S.
Rhinolophus ferrumequinum         ..S.....N. .......... .FE....... .RI.K.D.P. H........D
Rousettus aegyptiacus  Homo       ..S.....N. .......... .FE...L... .RI.Q...P. Y...A.....
sapiens (*)                       ..S....SN. .......... L.....Q... .QA.K...P. H.........
Piliocolobus tephrosceles (*)     ..S....SN. .......... L.....Q... .QA.K...P. H.........
Macaca mulatta (*)                ..S....SN. .......... L.....Q... .QA.K...P. H.........
Macaca nemestrina (*)             ..S....SN. .......... L.....Q... .QA.K...P. H.........
Choloepus didactylus              ..T.....N. .......... ......Q... .QA...Q.P. HR........
Anas platyrhynchos                ..A.....N. .......... .......... ..A...T.P. HT...T...A
Gallus gallus                     ..A.....N. .......... ......Q... ..A...T.P. H....T...A
```

```
                                          560        570        580        590        600
                                 ....|....| ....|....| ....|....| ....|....| ....|....|
Odocoileus virginianus (*)       AGQRLLQMLS LGKSEPWTLA LESIVGIKTM DVKPLLNYFE PLFTWLKEQN
Capreolus  Capreolus             .......... .......... .......... .......... ..........
Cervus elaphus                   .......... .......... .......... .......... ..........
Muntiacus muntjak                .......... .......... .......... .......... ..........
Oryx dammah (*)                  .........R .......... .......... .......... ..........
Nanger dama (*)                  .........R .......... ..N....... .......... ..........
Bubalus bubalis (*)              .........R .......... ..N....... .......... ..........
Bos taurus (*)                   .........R .......... ..N....... .......... ..........
Balaenoptera musculus (*)        .........H .......... ..N...V... .......... ..L.......
Tursiops truncatus (*)           .........H ........S. ..R...V... .......... ..L....G..
Sus scrofa (.)                   ...K...... .......... ..N...V... ......S... ..L....A..
Manis pentadactyla (*)           ...K...... ....K..... ..RV..T.N. ..R....... ..L.......
Felis catus (*)                  ..KK.....T ....K..... ..HV..E.K. N.T...K... ..........
Mustela putorius                 ...K.HE... ..R.K...F. ..RV..A... ..R....... ..........
Neogale vison                    ...K.HE... ..R.K...F. ..RV..A... ..R....... ..........
Canis lupus (.)                  ...K..E..K ....K...Y. ..IV..A.N. ..R....... ..........
Nyctereutes procyonoides (*)     ...K..E..K ....K...Y. ..IV..A.N. ..R....... ..........
Rhinolophus ferrumequinum        ..EK.H.... V...Q...SV .KDF..S.N. ..G...R... ..Y...T...
Rousettus aegyptiacus  Homo      ..KK.H.... ....K..... ....A.T.N. ..R....... ........K.
sapiens (*)                      ...K.FN..R .......... ..NV..A.N. N.R....... .......D..
Piliocolobus tephrosceles (*)    ...K..N..K .......... ..NV..A.N. N.R....... .......D..
Macaca mulatta (*)               ...K..N..K ..E....... ..NV..A.N. N.R....... .......D..
Macaca nemestrina  (*)           ...K..N..K .......... ..NV..A.N. N.R....... .......D..
Choloepus didactylus             ...K..N..K S.......A. ..HV..T.H. .......... .......D..
Anas platyrhynchos               ..GS.REL.K ..R.K...Q. ...LT.E.Y. NAT...H... ...N..QKN.
Gallus gallus                    ..GN.R.L.E ....K...Q. ...AT.E.Y. NAT...H... ...N..QKN.
```

```
                                      610        620        630        640        650
                               ....|....| ....|....| ....|....| ....|....| ....|....|
Odocoileus virginianus (*)     RNSFVGWSTE WTPYSDQSIK VRIS------ ---------- ---LKSALG-
Capreolus  Capreolus           .......... ...XXXXXXX XXXXXXXXXX XXXYEWNDNE MYLFR..VAY
Cervus elaphus                 K......... .......... ....LKSALG KNAYEWNDNE LYLFR.SVAY
Muntiacus muntjak              .......... .......... ....LKSALG KNAYEWNDNE MYLFR.SVAY
Oryx dammah (*)                .......... .......... ....LKSGLG KNAYEWNDNE MYLFR.SVAY
Nanger dama (*)                .......... .......... ....LKSALG KNAYEWNDNE MYLFR.SVAY
Bubalus bubalis (*)            .......... .......... ....LKAALG ENAYEWNDNE MYLFR.SVAY
Bos taurus (*)                 .......... .......... ....LKSALG ENAYEWNDNE MYLFQ.SVAY
Balaenoptera musculus (*)      ...S.....D .......... ....LKSALG EKAYEWNDNE MYLFR.SVAY
Tursiops truncatus (*)         .......R.D .....N.... ....LKSALG EKAYEWNDNE MYLFR.SVAY
Sus scrofa (.)                 G..S...N.D ....A..... ....LKSALG KEAYEWNDNE MYLFR.SIAY
Manis pentadactyla (*)         K......N.D .S..AA.... ....LKSALG EKAYEWNDSE MYLFR.SVAY
Felis catus (*)                .......N.D .R..A..... ....LKSALG DEAYEWNDNE MYLFR.SVAY
Mustela putorius               .......N.D .S..A..... ....LKSALG EKAYEWNDNE MYFFQ.SIAY
Neogale vison                  .......N.D .S..A..... ....LKSALG EKAYEWNDNE MYFFQ.SIAY
Canis lupus (.)                .......N.D .S..A..... ....LKSALG EKAYEWNNNE MYLFR.SIAY
Nyctereutes procyonoides (*)   .......N.D .S..A..... ....LKSALG EKAYEWNNNE MYLFR.SIAY
Rhinolophus ferrumequinum      .K.....N.D .S..A..... ....LKSALG EKAYEWNNNE MYLFR.SVAY
Rousettus aegyptiacus   Homo   .........D .S...G.... ....LKAALG EKAYEWNDNE MYLF..SIAY
sapiens (*)                    K........D .S..A..... ....LKSALG DKAYEWNDNE MYLFR.SVAY
Piliocolobus tephrosceles (*)  K........D .S..A..... ....LKSALG DKAYEWNDNE MYLFR.SVAY
Macaca mulatta (*)             K........D .S..A..... ....LKSALG DKAYEWNDNE MYLFR.SVAY
Macaca nemestrina  (*)         K........D .S..A..... ....LKSALG DKAYEWNDNE MYLFR.SVAY
Choloepus didactylus           ..VP.....D GS.DA..-.. ....LKSALG DKAYEWNDNE MYLFR.SVAY
Anas platyrhynchos             SGRYI..N.D .....ENA.. ....LKAAG- -QTYEWNKSE LFLF..TIAY
Gallus gallus                  SGRSI..N.D ......NA.. ....LKAALG DDAYVWDASE LFLF..SIAY
```

	660	670	680	690	700
	....|....|	....|....|	....|....|	....|....|	....|....|
Odocoileus virginianus (*)	----------	----------	---KNADANC	PFVWCVPPVS	HLVAIVIRSA
Capreolus Capreolus	AMRKYFLGER	NETIPFGEEN	VWVSDKKPRI	S.KFF.TS.N	NVSD..P.TE
Cervus elaphus	AMRKYFLKKR	NETIPFGEEN	VWVSDKKPRI	S.KFF.TSPN	NVSD.IP.TE
Muntiacus muntjak	AMRKYFLKKR	NETIPFGEEN	VWVSDKKPRI	S.KFF.TSPN	NVSD.IP.TE
Oryx dammah (*)	AMRKYFFKDR	NETIPFGEEN	VWVSDKKPRI	S.KFF.TSPN	NVSD.IP.TE
Nanger dama (*)	AMRRYFFEAS	NETIPFGAEN	VWVSDKKPRI	S.KFF.TSPN	NVSD.IP.TE
Bubalus bubalis (*)	AMRKYFSEAR	NETVLFGEDN	VWVSDKKPRI	S.KFF.TSPN	NVSD.IP.TE
Bos taurus (*)	AMRKYFSEAR	NETVLFGEDN	VWVSDKKPRI	S.KFF.TSPN	NVSD.IP.TE
Balaenoptera musculus (*)	AMREYFSKVR	NETIPFGEKD	VWVSDLKPRI	S.NFF.TTPK	NVSD.IS.TE
Tursiops truncatus (*)	AMREYFSKVR	NKTIPFGEKD	VWVSDLKPRI	S.NFF.TSPK	NMSD.IP.TE
Sus scrofa (.)	AMRNYFSSAK	NETIPFGAED	VWVSDLKPRI	S.NFF.TSPA	NMSD.IP..D
Manis pentadactyla (*)	AMREYFSKFK	KQTIPFEEES	VRVSDLKPRV	S.IFF.TLPK	NVS.VIP.AE
Felis catus (*)	AMREYFSKVK	NQTIPFVEDN	VWVS.LKPRI	S.NFF.TASK	NVSDVIP..E
Mustela putorius	AMREYFSKVK	NQTIPFVGKD	VRVSDLKPRI	S.NFI.TSPE	NMSD.IP.AD
Neogale vison	AMREYFSKVK	KQTIPFVDKD	VRVSDLKPRI	S.NFI.TSPE	NMSD.IP.AD
Canis lupus (.)	AMRQYFSEVK	NQTIPFVEDN	VWVSDLKPRI	S.NFS.TSPG	NVSD.IP.TE
Nyctereutes procyonoides (*)	AMRQYFSEVK	NQTIPFVEDN	VWVSDLKPRI	S.NFF.TSPG	NVSD.IP.TE
Rhinolophus ferrumequinum	AMREYFLKTK	NQTILFGEED	VWVS.LKPRI	S.NFY.TSPR	N.SD.IP.PE
Rousettus aegyptiacus Homo	SLREYFLKVK	NLTIPFGEED	VWVSDLKPRI	S.NFF.TSPQ	NVSEFIP.TE
sapiens (*)	AMRQYFLKVK	NQMILFGEED	VRVA.LKPRI	S.NFF.TAPK	NVSD.IP.TE
Piliocolobus tephrosceles (*)	AMRKYFLEIK	HQTILFGEED	VRVADLKPRI	S.NFY.TAPK	NVSD.IP.TE
Macaca mulatta (*)	AMRTYFLEIK	HQTILFGEED	VRVADLKPRI	S.NFY.TAPK	NVSD.IP.TE
Macaca nemestrina (*)	AMRTYFLEIK	HQTILFGEED	VRVADLKPRI	S.NFY.TAPK	NVSD.IP.TE
Choloepus didactylus	AMREYFLKVK	NQTILFGEED	VQVSELKPRI	S.IFV.SAPN	NTSD.IP.TE
Anas platyrhynchos	AMRTYFAQ-K	QQLIDFEATD	VHVSEETQRV	S.YIT.SMPG	NASN..PKAD
Gallus gallus	AMRKYFAKEK	EQNVDFQVTD	IHVGEETQRV	S.YLT.SMPG	NVSD..P.AD

```
                                              710        720        730        740        750
                                      ....|....| ....|....| ....|....| ....|....| ....|....|
Odocoileus virginianus (*)            VT_________ __________ __________ __________ _________V
Capreolus  Capreolus                  .EEAIRLSRG RINDAFQLDD NSLEFLGIQP TLGPPYEPPV TIWLIIFGV.
Cervus elaphus                        .ENAIRLSRY RINDAFQLDD NSLEFLGIQP TLGPPYKPPV TIWLIIFGV.
Muntiacus muntjak                     .ENAIRLSRD RINDAFQLDD NSLEFLGIQP TLGPPYEPPV TIWLIIFGV.
Oryx dammah (*)                       .ENAIRLSRD RINDAFQLDD NSLEFLGIQP TLGPPYEPPV TIWLIIFGV.
Nanger dama (*)                       .ENAIRLSRD RINDAFQLDD NSLEFLGIQP TLGPPYEPPV TIWLIIFGV.
Bubalus bubalis (*)                   .ENAIRLFRG RINDVFQLDD NSLEFLGIQP TLRPPYEPPV TIWLIIFGV.
Bos taurus (*)                        .ENAIRLSRD RFNDVFQLDD NSLEFLGIQP TLGPPYEPPV TIWLIIFGV.
Balaenoptera musculus (*)             .EEAIRMSRG RINDAFRLDD NSLEFLGIQP TLGPPYEPPV TIWLIIFGA.
Tursiops truncatus (*)                .EEAIRMSRG RINDAFRLDD SSLEFLGVQP TLAPPYEPPV TVWLIIFGV.
Sus scrofa (.)                        .EKAISMSRS RINDAFRLDD NTLEFLGIQP TLGPPDEPPV TVWLIIFGV.
Manis pentadactyla (*)                .EEAIRMSRS RINDVFRLDD NSLEFLGIQP TLEPPYQPPV TIWLIVFGV.
Felis catus (*)                       .EEAIRMSRS RINDAFRLDD NSLEFLGIQP TLSPPYQPPV TIWLIVFGV.
Mustela putorius                      .EEAIRKSRG RINDAFRLDD NSLEFLGIQP TLEPPYQPPV TIWLIVFGV.
Neogale vison                         .EEAIRKSRG RINDAFRLDD NSLEFLGIQP TLEPPYQPPV TIWLIVFGV.
Canis lupus (.)                       .EEAIRMYRS RINDVFRLDD NSLEFLGIQP TPGPPYEPPV TIWLIVFGV.
Nyctereutes procyonoides (*)          .EEAIRMYRS RINDVFRLDD NSLEFLGIQP TLGPPYEPPV TIWLIVFGV.
Rhinolophus ferrumequinum             .EGAIRMSRS RINDAFRLDD NSLEFLGIQP TLGPPYQPPV TIWLIVFGV.
Rousettus aegyptiacus  Homo           .EGAIRMSRS RINDAFRLDD DTLEFLGIEP TLGTPYQPPV TIWLIVFGV.
sapiens (*)                           .EKAIRMSRS RINDAFRLND NSLEFLGIQP TLGPPNQPPV SIWLIVFGV.
Piliocolobus tephrosceles (*)         .EEAIRLSRS RINDAFRLND DSLEFLGIQP TLAPPYQPPV TIWLIVFGV.
Macaca mulatta (*)                    .EEAIRISRS RINDAFRLND NSLEFLGIQT TLAPPYQSPV TTWLIVFGV.
Macaca nemestrina  (*)                .EEAIRISRS RINDAFRLND NSLEFLGIQT TLAPPYQSPV TTWLIVFGV.
Choloepus didactylus                  .EKAISMSRG RINDAFRLDD NTLEFVGIYP TLAPPYEPPV VIWLVVFGVI
Anas platyrhynchos                    .ESAISMSRG RINEAFGLDD DTLEFVGIIP TLAAPYEPPV TIWLIIFGV.
Gallus gallus                         .EKAIRMSRG RISEAFRLDD NTLEFDGIVP TLATPYKPPV TIWLILFGV.
```

Species	....|....|	760 ....|....|	770 ....|....|	780 ....|....|	790 ....|....| 800
Odocoileus virginianus (*)	SQCCVQATLV	LLNPGPK---	-----VPEE-	----------	----------
Capreolus Capreolus	MGVV.LGIV.	.IFT.IRDRR	KKNQASS..N	PYG-------	SVDLNK--GE
Cervus elaphus	MGVV.IGIV.	.IFT.IRDRR	KKNQASS..N	PYG-------	SVDLNK--GE
Muntiacus muntjak	MGVV.IGIV.	.IFT.IRDRR	KKNQVSS..N	PYG-------	SVDLNK--GE
Oryx dammah (*)	MGVV.IGIVG	.IFT.IRDQR	KKNQASS..N	PYG-------	SVDLNK--GE
Nanger dama (*)	MGVV.IGIV.	.IFT.IQDRR	KHDNDGLQND	ENLRVQQQAV	KVDIPR--NS
Bubalus bubalis (*)	MGVV.IGII.	.IFT.IRDRR	KKNQASS..N	PYG-------	SVDLNK--GE
Bos taurus (*)	MGVV.IGIV.	.IFT.IRNRR	KKNQASS..N	PYG-------	SVDLNK--GE
Balaenoptera musculus (*)	MGVV.IGIA.	.IFT.IRDRR	EKSQASS..N	PYI-------	SMDLSK--GE
Tursiops truncatus (*)	MGVV.IGIV.	.IFT.IRDRR	KKNQASS..N	PYG-------	SVGLSK--GE
Sus scrofa (.)	MGLV.VGIV.	.IFT.IRDRR	KKKQASS..N	PYG-------	SMDLSK--GE
Manis pentadactyla (*)	MGVI.VGIV.	.IFT.IRDRK	KKNQARS.QN	PYA-------	SVDLSK--GE
Felis catus (*)	MGVV.VGIVL	.IVS.IRNRR	KNNQARS..N	PYA-------	SVDLSK--GE
Mustela putorius	MGVV.VGIFL	.IFS.IRNRR	KNNQARS..N	PYA-------	SVDLSK--GE
Neogale vison	MGVV.VGIFL	.IFS.IRNRR	KNNQARS..N	PYA-------	SVDLSK--GE
Canis lupus (.)	MGVV.VGIVL	.IFS.IRNRR	KNDQARG..N	PYA-------	SVDLSK--GE
Nyctereutes procyonoides (*)	MGVV.VGIVL	.IFS.IRNRR	KNDQARG..N	PYA-------	SVDLSK--GE
Rhinolophus ferrumequinum	MAVV.VGIV.	.IIT.IRDRR	KKDQARS..N	PYS-------	SVDLSK--GE
Rousettus aegyptiacus	MGLV.VGIVL	.IFV.IRDRR	KKNQERS..N	PYS-------	SVDLSK--GE
Homo sapiens (*)	MGVI.VGIVI	.IFT.IRDRK	KKNKARSG.N	PYA-------	SIDISK--GE
Piliocolobus tephrosceles (*)	MGVI.AG.V.	.IFT.IRDRK	KKNQARS..N	PYA-------	SIDISK--GE
Macaca mulatta (*)	MGVI.AGIV.	.IFT.IRDRK	KKNQARS..N	PYA-------	SIDINK--GE
Macaca nemestrina (*)	MGVI.AGIV.	.IFT.IRDRK	KKNQARS..N	PYA-------	SIDINK--GE
Choloepus didactylus	MSVI.IGIVL	.IFT.IRERK	KNRQASGQ.N	PYA-------	SVDLSK--GE
Anas platyrhynchos	ISLV.IGVI.	.IVS.QRDRK	KKAKGRER.A	ESN------C	EVNPYDDDGR
Gallus gallus	MSLI.IGVI.	.IIT.QRDKR	KKARGRAN.A	GSN------C	EVNPYDEDGR

	810<br>....|....| ....|
Odocoileus virginianus (*)	---------- -----
Capreolus Capreolus	NNSGFQNTDD VQTSL
Cervus elaphus	NNSGFQNTDD VQTSL
Muntiacus muntjak	NNSGFQNTDD VQTSL
Oryx dammah (*)	NNSGFQNTDD VQTSL
Nanger dama (*)	LKATVPFSNS PEKLK
Bubalus bubalis (*)	NNSGFQNTDD VQTSL
Bos taurus (*)	NNSGFQNIDD VQTSL
Balaenoptera musculus (*)	NNSGFQNSGD VHTSF
Tursiops truncatus (*)	NNPGFQNSDD VQTSF
Sus scrofa (.)	SNSGFQNGDD IQTSF
Manis pentadactyla (*)	NNPGFQNVDD VQTSF
Felis catus (*)	NNPGFQHADD VQTSF
Mustela putorius	NNPGFQNVDD VQTSF
Neogale vison	NNPGFQNVDD VQTSF
Canis lupus (.)	NNPGFQSGDD VQTSF
Nyctereutes procyonoides (*)	NNPGFQNVDD AQTSF
Rhinolophus ferrumequinum	NNPGFQNGDD VQTSF
Rousettus aegyptiacus Homo	NNAGFQNNDD VQTSF
sapiens (*)	NNPGFQNTDD VQTSF
Piliocolobus tephrosceles (*)	NNPGFQNTDD VQTSF
Macaca mulatta (*)	NNPGFQNTDD VQTSF
Macaca nemestrina (*)	NNPGFQNTDD VQTSF
Choloepus didactylus	NNPGFQNSDD IQTSF
Anas platyrhynchos	SNKGFELSDE TQTSF
Gallus gallus	SNKGFEQSEE TQTSF

Figure S1. Multiple sequence alignment of the full-length ACE2 protein sequences for the species characterized for the species characterized in [7] and additional deer species included from GenBank (GCA_000751575.1, XP_037678579.1, XP_012949915.3, QEQ50331.1, KAB0345583.1, XP_043752042.1, XP_036696353.1). Alignments were performed using ClustalW Multiple Alignment in BioEdit version 7.2.5. The names of species with (*) correspond to those with high or moderate susceptibility to SARS-CoV-2 while (.) correspond to species with low susceptibility to SARS-CoV-2. The positions highlighted in red boxes are considered as known binding residues and predictions of SARS-CoV-2 S-binding propensity [7]. Positions K31, E35, N53, M82, N90, N323 and K354 (this last two shifted one amino acid from [7]) are reported as binding hot spots residues. The high variability in the aligned sequences of ACE2 from position 600 is delimited with a green line.
